# PCA-based spatial domain identification with state-of-the-art performance

**DOI:** 10.1093/bioinformatics/btaf005

**Published:** 2025-01-07

**Authors:** Darius P Schaub, Behnam Yousefi, Nico Kaiser, Robin Khatri, Victor G Puelles, Christian F Krebs, Ulf Panzer, Stefan Bonn

**Affiliations:** Institute of Medical Systems Bioinformatics, Center for Biomedical AI (bAIome), Center for Molecular Neurobiology (ZMNH), University Medical Center Hamburg-Eppendorf, Hamburg 20246, Germany; III Department of Medicine, University Medical Center Hamburg-Eppendorf, Hamburg 20246, Germany; Institute of Medical Systems Bioinformatics, Center for Biomedical AI (bAIome), Center for Molecular Neurobiology (ZMNH), University Medical Center Hamburg-Eppendorf, Hamburg 20246, Germany; German Center for Child and Adolescent Health (DZKJ), Partner Site Hamburg, University Medical Center Hamburg-Eppendorf, Hamburg 20246, Germany; Institute of Medical Systems Bioinformatics, Center for Biomedical AI (bAIome), Center for Molecular Neurobiology (ZMNH), University Medical Center Hamburg-Eppendorf, Hamburg 20246, Germany; III Department of Medicine, University Medical Center Hamburg-Eppendorf, Hamburg 20246, Germany; Institute of Medical Systems Bioinformatics, Center for Biomedical AI (bAIome), Center for Molecular Neurobiology (ZMNH), University Medical Center Hamburg-Eppendorf, Hamburg 20246, Germany; III Department of Medicine, University Medical Center Hamburg-Eppendorf, Hamburg 20246, Germany; Hamburg Center for Kidney Health (HCKH), University Medical Center Hamburg-Eppendorf, Hamburg 20246, Germany; Department of Clinical Medicine, Aarhus University, Aarhus 8200, Denmark; Department of Pathology, Aarhus University Hospital, Aarhus 8200, Denmark; III Department of Medicine, University Medical Center Hamburg-Eppendorf, Hamburg 20246, Germany; Hamburg Center for Kidney Health (HCKH), University Medical Center Hamburg-Eppendorf, Hamburg 20246, Germany; Hamburg Center for Translational Immunology (HCTI), University Medical Center Hamburg-Eppendorf, Hamburg 20246, Germany; III Department of Medicine, University Medical Center Hamburg-Eppendorf, Hamburg 20246, Germany; Hamburg Center for Kidney Health (HCKH), University Medical Center Hamburg-Eppendorf, Hamburg 20246, Germany; Hamburg Center for Translational Immunology (HCTI), University Medical Center Hamburg-Eppendorf, Hamburg 20246, Germany; Institute of Medical Systems Bioinformatics, Center for Biomedical AI (bAIome), Center for Molecular Neurobiology (ZMNH), University Medical Center Hamburg-Eppendorf, Hamburg 20246, Germany; German Center for Child and Adolescent Health (DZKJ), Partner Site Hamburg, University Medical Center Hamburg-Eppendorf, Hamburg 20246, Germany; Hamburg Center for Kidney Health (HCKH), University Medical Center Hamburg-Eppendorf, Hamburg 20246, Germany; Hamburg Center for Translational Immunology (HCTI), University Medical Center Hamburg-Eppendorf, Hamburg 20246, Germany

## Abstract

**Motivation:**

The identification of biologically meaningful domains is a central step in the analysis of spatial transcriptomic data.

**Results:**

Following Occam’s razor, we show that a simple PCA-based algorithm for unsupervised spatial domain identification rivals the performance of ten competing state-of-the-art methods across six single-cell spatial transcriptomic datasets. Our reductionist approach, NichePCA, provides researchers with intuitive domain interpretation and excels in execution speed, robustness, and scalability.

**Availability and implementation:**

The code is available at https://github.com/imsb-uke/nichepca.

## 1 Introduction

Single-cell spatial transcriptomics (ST) extends our understanding beyond single-cell sequencing by revealing information about both individual cells and cell collectives. Similar to categorizing distinct cell types, these collectives can be classified into functionally or phenotypically distinct groups, known as spatial domains or niches. This allows us to describe disease pathology and dynamics in terms of cell–cell interactions and domain-specific cell-type distributions. Accurately identifying spatial domains is thus crucial to leverage the full potential of ST data. In recent years, many sophisticated methods have been developed to identify tissue domains from ST data in an unsupervised manner (Bhuva *et al.* 2024, Hu *et al.* 2024, Yuan *et al.* 2024), spanning from graph neural network-based (Cang *et al.* 2021, Hu *et al.* 2021, Dong and Zhang 2022, Li *et al.* 2022, Ren *et al.* 2022, Long *et al.* 2023, Liu *et al.* 2024) to Bayesian methods (Zhao *et al.* 2021, Li and Zhou 2022). These methods generally follow the same sequence of high-level steps: beginning with graph construction, then neighborhood embedding, and finally clustering. We call this the neighborhood embedding paradigm. Within this paradigm, most methods try to achieve superior domain identification performance by introducing complexities in one or more of the underlying substeps, e.g. by using graph neural networks as non-linear neighborhood embedding functions.

In this work, we investigate how much complexity is necessary for a model to achieve state-of-the-art spatial domain identification performance. Following Occam’s razor, we present a simple PCA-based workflow, NichePCA, and compare its domain identification performance to competing, more complex methods. The algorithm relies on only four steps: First, a k-nearest-neighbor graph is constructed using the segmented spatial data, with nodes representing cells and edges representing spatial cell adjacency. Second, the gene expression is normalized and log-transformed per cell and the mean gene expression of each cell and its neighbors is calculated. Third, PCA is performed on the aggregated gene expression counts to obtain a low-dimensional representation. Fourth, the spatial domains are identified using Leiden clustering (Traag *et al.* 2019). Note that the only difference between NichePCA and the common single-cell transcriptomic workflow for cell type identification is the spatial aggregation per cellular neighborhood.

## 2 Materials and methods

### 2.1 NichePCA

First, we normalize the raw ST data to sum to their medians and then apply a log-transformation, respectively, using the *normalize_total* and *log1p* functions of the Scanpy (v1.9.8) Python package (Wolf *et al.* 2018, Virshup *et al.* 2023). Next, we create a spatial graph with nodes representing cells and edges representing spatial adjacency between cells. To define cell adjacencies, either a distance threshold or a specified number *K* of nearest neighbors (KNN) can be used. By default, we use the KNN approach. Importantly, we ensure the graph is undirected by adding missing edges to the directed ones. The resulting cell neighborhood graph also includes self-loops. Then, for each cell, we calculate the mean gene expression of its neighbors, including the cell itself, and perform PCA on these aggregated gene expression vectors. Finally, the spatial domains are identified using Leiden clustering. In total, NichePCA depends on three hyperparameters: the number of nearest neighbors *K*, the number of principal components (PCs), and the Leiden resolution. However, throughout all our experiments, we kept the number of PCs fixed to 30.

### 2.2 Existing spatial clustering methods

Here we provide a brief overview of existing spatial clustering methods, including Banksy (Singhal *et al.* 2024), SpatialPCA (Shang and Zhou 2022), BASS (Li and Zhou 2022), and SCAN-IT (Cang *et al.* 2021).

#### 2.2.1 Banksy

This method uses three different cell- and neighborhood-level representations, including the raw gene expression of the cell, the aggregated raw expression of the neighboring cells (based on KNN), and a gene expression gradient in the cellular neighborhood. It concatenates these three representations into a single matrix and then applies PCA followed by Leiden clustering to obtain domain clusters.

#### 2.2.2 SpatialPCA

This is a spatially aware dimension reduction method aimed at inferring a low-dimensional representation of the spatial transcriptome data. In particular, SpatialPCA factorizes the gene expression matrix into a pair of matrices: the loading matrix and hidden factors. To reflect the spatial information, SpatialPCA assumes that each hidden factor is derived from a multivariate normal distribution function where its covariance matrix models the correlation among the spatial locations. Lastly, it uses Louvain clustering to determine the spatial domains.

#### 2.2.3 SCAN-IT

This is a deep learning-based method for spatial domain identification. It first builds a spatial network where nodes represent cells. Next, it uses a graph convolutional neural network to represent cells in an embedding space. Finally, it applies Leiden clustering on cells in the embedding space to identify spatial domains.

#### 2.2.4 BASS

This method employs a hierarchical Bayesian approach for spatial domain segmentation. It models three key probabilities: the likelihood of observing specific gene expression in a cell, conditional on its type; the likelihood that a cell belongs to a particular type, conditioned on its spatial domain; and the likelihood of a spatial domain as a function of the neighborhood graph. Using this model, the spatial domains are determined through Bayesian inference.

### 2.3 Datasets and ground truth annotations

We followed the same preprocessing strategy for all datasets, namely removing all cells with <10 counts and removing all genes expressed in <5 cells. A detailed overview of all datasets and their sources can be found in Supplementary Tables S1 and S2.

#### 2.3.1 Dataset 1

This dataset was acquired via MERFISH technology (Moffitt *et al.* 2018) and published in 2018. The number of measured genes is 155 and cells were segmented using a seeded watershed algorithm applied to the DAPI and total mRNA co-stains. Originally the dataset contains 12 samples of which only five were annotated with spatial domain labels (Li and Zhou 2022). These samples contain between 5000 and 6000 cells each. We obtained the annotated dataset from the resource provided by Yuan *et al.* (2024) (Supplementary Table S1).

#### 2.3.2 Dataset 2

This dataset contains STARmap measurements for three mouse medial prefrontal cortex samples with 166 genes and one sample of the mouse visual cortex measured with Starmap technology and 1020 genes (Wang *et al.* 2018). Cell segmentation was performed by manual cell nuclei identification and a subsequent cell body segmentation based on a Nissl staining. The layers were previously annotated by an expert (Li and Zhou 2022). The four samples contain between 1000 and 1200 cells each. We downloaded the data from the resource provided by Yuan *et al.* (2024) (Supplementary Table S1).

#### 2.3.3 Dataset 3

This dataset was measured using the BaristaSeq technology and contains three samples derived from the mouse cortex (Chen *et al.* 2018). The number of measured genes is 79 and each sample contains between 1500 and 2000 cells. Spatial domains have been manually annotated (Long *et al.* 2023). We downloaded the data from the resource provided by Yuan *et al.* (2024) (Supplementary Table S1).

#### 2.3.4 Dataset 4

This publicly available dataset contains three mouse brain samples measured with Xenium technology by the manufacturer 10× Genomics. The number of unique genes is 248 and cells were segmented by 10× Genomics using their proprietary cell segmentation pipeline. Each sample contains about 150 000 cells. The dataset has been recently annotated by Bhuva *et al.* (2024) using the Allen Brain Reference Atlas (Wang *et al.* 2020). They performed a transcript-level annotation of spatial domains, which allowed us to easily consider two different cases per sample, one only using the transcripts measured inside the nuclei and another one using all transcripts measured inside the segmented cells (Supplementary Fig. S2). We removed all dissociated cells, by defining a cell graph based on a distance of 60 microns and removing all cells that were not part of the largest connected component. We downloaded the data from the resource provided by Bhuva *et al.* (2024).

#### 2.3.5 Dataset 5

This publicly available dataset was measured with the MERFISH technology by the manufacturer Vizgen. The number of measured genes is 483 and cells were segmented by Vizgen with the default Vizgen segmentation pipeline. Since the dataset lacks ground truth domain annotations we developed an automated annotation workflow similar to the one by Bhuva *et al.* (2024) (see next section). Originally the dataset contains nine samples derived from the mouse brain of which we only consider the four most symmetric ones, to ensure the quality of our automated domain annotation workflow. The four samples each contain about 80 000 cells. The links to the raw data can be found in Supplementary Table S1.

#### 2.3.6 Dataset 6

This dataset contains 31 mouse brain samples measured with MERFISH technology (Allen *et al.* 2023). The number of unique genes is 374 and cells were segmented by the authors using Cellpose (Stringer *et al.* 2021). The spatial domains were annotated based on the manual cell type annotations. First, they overclustered each cell’s neighborhood cell type composition after PCA and then manually merged subclusters to obtain eight domain clusters. We downloaded the data from CELLxGENE using the link provided in Supplementary Table S1.

#### 2.3.7 Dataset 7

This dataset is part of the BICCN 2.0 atlas and contains four different sub-datasets, all measuring the mouse brain via MERFISH technology (Zhang *et al.* 2023). We refer to these as Datasets 7.1 to 7.4. The number of unique genes for these datasets is 1122 and cells were segmented by the authors using Cellpose 2.0 (Pachitariu and Stringer 2022). Dataset 7.1 contains 50 samples and 1 006 300 cells. Dataset 7.2 contains 50 samples and 982 842 cells. Dataset 7.3 contains 22 samples and 1 566 842 cells. Dataset 7.4 contains 3 samples and 162 361 cells. Datasets 7.1 and 7.2 originally contained more samples but we restricted our analysis to a random subset of 50 samples each. The spatial domains were hierarchically annotated based on the manual cell type annotations. We followed the tutorial provided at https://alleninstitute.github.io to download and preprocess the data (Supplementary Table S1).

### 2.4 Automated annotation workflow

To annotate the ground truth spatial domains for Dataset 5, we used the Allen Brain Reference Atlas (Wang *et al.* 2020), which provides a 3D reference volume of an entire mouse brain. First, an axial slice that best matches the anatomical regions of the DAPI image was selected manually via the Allen Python SDK for every individual MERFISH slice. Next, the reference slices and DAPI images were downscaled to a resolution of 25 microns per pixel to reduce computational cost. We then used the Elastix (Klein *et al.* 2010) toolkit and the pyelastix Python wrapper to register the images in a two-step process. First, a rigid transformation was computed that allows only translational and rotational transformation of the moving image. Subsequently, an affine transformation was applied that allows shearing and stretching. The second step is particularly important since the orientations of the utilized MERFISH slices did not always perfectly match the axial view of the reference volume and were not perfectly symmetric. Mutual information was used as a similarity metric between the two images and stochastic gradient descent-based optimization was performed until convergence. Finally, the annotated axial slice corresponding to the DAPI image was transformed according to the previously computed parameters, and MERFISH cell coordinates were assigned based on the overlap of their respective centroids with a specific annotation ID.

### 2.5 Evaluation metrics

To evaluate and compare the performances of the different spatial domain identification methods in recognizing the “ground-truth” labels (we also refer to as class), we considered the following evaluation metrics:

#### 2.5.1 Normalized mutual information

Normalized mutual information (NMI) is a measure used to evaluate the similarity between two data clusterings (i.e. the model-based and ground-truth) based on their mutual information. The value is normalized between 0 and 1. A score of 1 indicates perfect clustering correspondence, while a score of 0 indicates no clustering correspondence. We implemented NMI via the *normalized_mutual_info_score* function from the scitkit-learn (v1.3.0) Python package.

#### 2.5.2 Homogeneity score

Homogeneity score (HOM) is a measure indicating the purity of clusters with respect to the ground-truth labels. It quantifies whether each cluster predominantly consists of data points from a single class. A HOM of 1 indicates perfect homogeneity, while a score of 0 indicates poor homogeneity. We implemented HOM via the *homogeneity_score* function from the scitkit-learn (v1.3.0) Python package.

#### 2.5.3 Completeness score

Completeness score (COM) is a measure to evaluate the extent to which all members of a given class are assigned to the same cluster recognized by the models. A COM of 1 indicates that the model perfectly clusters all members of each class, while a score of 0 suggests that members of the same class are scattered across different clusters. We implemented COM via the *completeness_score* function from the scitkit-learn (v1.3.0) Python package.

### 2.6 Benchmarking workflow and setup

On Datasets 1 to 3, we reused the quantitative results provided by Yuan *et al.* (2024) and compared them against NichePCA, Banksy, and SpatialPCA using a similar benchmarking workflow. In particular, the number of domain clusters identified with each method was chosen to fit the ground truth annotations for each dataset. Additionally, for NichePCA and Banksy we varied the number of nearest neighbors in the range from 5 to 20 and chose the value with the best performance across all datasets and all three metrics. For SpatialPCA, we tested different kernel bandwidths in the range from 0.1 to 0.5, again selecting the value showing the best performance. To produce a visual plot of the domains identified by SCAN-IT for a selected sample from Dataset 1 we used the same settings as Yuan *et al.* (2024).

On Datasets 4 and 5, we only tested the top three performing methods besides NichePCA: Banksy, SCAN-IT, and SpaceFlow. We were not able to run BASS or SpatialPCA within a memory budget of 128 GB, which we deem reasonable to ensure practical usability. For each method and sample of Datasets 4 and 5, we varied the number of nearest neighbors for spatial graph construction between 5 and 29 and the resolution for Leiden clustering (used by all four methods by default) between 0.1 and 2.0 with a step size of 0.1 while restricting the maximum number of clusters to 60. Then we selected the best-performing resolution across the average of all metrics for each method, each sample, and each number of the nearest neighbors setting. Lastly, we selected the number of nearest neighbors per method across both datasets such that the average performance across all metrics and the previously determined resolution settings is maximal. The corresponding metrics for these runs were then compared between methods. Since for Dataset 5, some of the cells could not be assigned to any of the atlas reference domains we ignored them during metric calculation.

### 2.7 Multi-sample spatial domain identification

To obtain coherent spatial clusters across multiple samples, we utilized the Python implementation of Harmony (Korsunsky *et al.* 2019) accessible via Scanpy (v1.9.8). Importantly, the graph construction and feature aggregation were performed separately for each sample while the PCA was applied across all samples. Finally, we performed Leiden clustering to obtain the spatial domains. We applied this workflow to Dataset 6 and considered the same number of nearest neighbors as determined in our experiments on Datasets 4 and 5. We selected a Leiden resolution of 0.15, which produced a similar number of clusters per sample as in the ground truth annotations. For visualization, we matched the cluster colors with their corresponding domains in the ground truth annotations (Fig. 3b). BASS also supports coherent spatial domain identification across multiple samples, but we were not able to execute it within a memory budget of 128 GB, which we consider a reasonable limit to ensure practical usability.

### 2.8 Large-scale multi-sample spatial domain identification

We applied the same workflows as for our previous multi-sample spatial domain identification experiment to all sub-datasets of Dataset 7. Specifically, we selected the same number of nearest neighbors as determined in our experiments on Datasets 4 and 5. We selected the Leiden resolution per sub-dataset so that the total number of clusters is <60 and the NMI calculated across all samples is maximal. Additionally, we calculated the NMI score per sample (Supplementary Fig. S5).

### 2.9 Scalability analysis

We measured compute times and memory usage for NichePCA, Banksy, SpaceFlow, and SCAN-IT on multiple subsampled versions of a selected sample of Dataset 4 with cell numbers ranging from 5000 to 155 000. We executed each method 10 times per subsample and chose the median value for both measures. We also tracked the clustering time for SCAN-IT, SpaceFlow, and NichePCA, as these methods use a separate Leiden clustering step. All methods were executed in a single thread on an AMD EPYC 7742 64-core processor.

### 2.10 Interpretability

On MERFISH brain data, we measured the variation explained by the PCs and assessed the feature loadings of the first PC using Scanpy (v1.9.8). The PC coordinates of the 54 cluster centroids were computed by taking the mean of the 30 PCs, resulting in a 54 × 30 matrix. This matrix was multiplied with the PCA feature loadings (a matrix of dimension 30 × 483), resulting in a 54 × 483 matrix with gene contributions to each cluster.

### 2.11 Ablation study

We conducted an ablation study to understand better which components of our computational pipeline are critical for identifying spatial domains. Therefore, we considered all possible variations involving the same or fewer steps than the original NichePCA, namely median normalization, log-transformation, mean neighborhood aggregation, and PCA. Since we can presume that the aggregation step is critical we only considered one algorithm without it. For comparability, we applied all ablations and NichePCA on Datasets 1–3 using the same number of nearest neighbors (*K *=* *7).

### 2.12 Computational resources

All analyses were performed on a server with an AMD EPYC 7742 central processing unit (2.25 GHz, 512 MB L3 cache, 64 CPU cores in total), and 128 GB of memory.

## 3 Results

**Figure 1.**
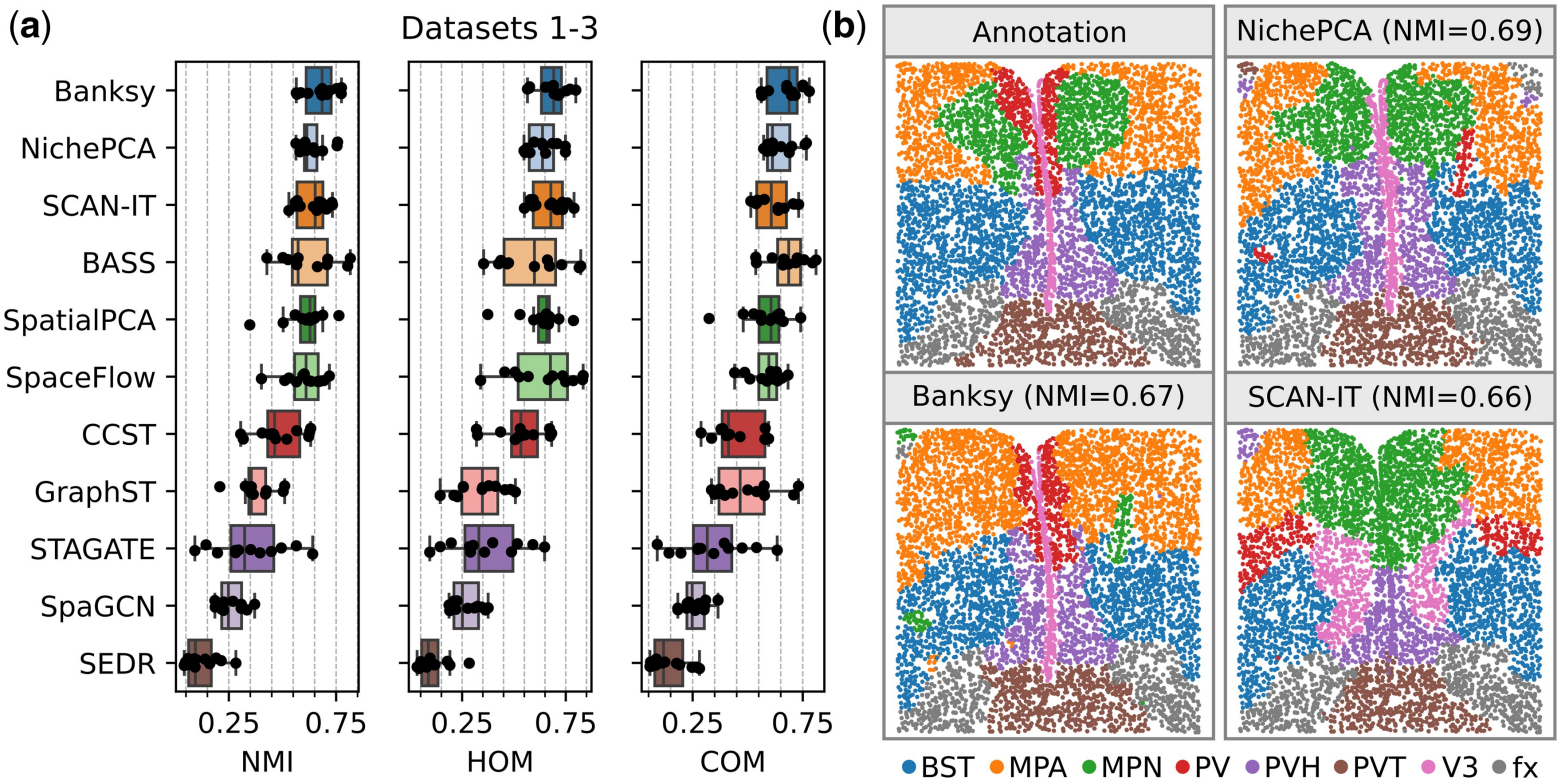
Quantitative performance evaluation on small-scale data (Datasets 1–3). (a) Performance of different spatial domain identification methods on Datasets 1–3 in terms of NMI, HOM, and COM scores. The dots indicate the performance per sample. For SCAN-IT, BASS, SpaceFlow, CCST, GraphST, STAGATE, SpaGCN, and SEDR we adopted the results from Yuan *et al.* (2024). For NichePCA, Banksy, and SpatialPCA, we followed an analogous benchmarking setup as in Yuan *et al.* (2024) to ensure comparability (see Section 2). (b) Exemplary domain identification results for NichePCA, Banksy, and SCAN-IT on a selected sample of Dataset 1 (MERFISH) and corresponding ground truth annotations. In brackets, we provide the performance in terms of NMI for each method on this specific sample.

We first assessed the performance of NichePCA by following the recently published spatial domain identification benchmark of Yuan *et al.* (2024). In their study, 13 spatial clustering methods were evaluated on multiple real and simulated single-cell ST datasets, each with expert-validated ground truth annotations. We extended this list by two additional methods, SpatialPCA (Shang and Zhou 2022) and Banksy (Singhal *et al.* 2024). We considered three of Yuan *et al.*’s real datasets (our Datasets 1–3) that were generated using MERFISH (Chen *et al.* 2015), BaristaSeq (Chen *et al.* 2018), and STARmap (Wang *et al.* 2018) technologies (see Section 2). The performance of identifying spatial domains was evaluated against the ground truth annotations using the Normalized Mutual Information (NMI), Homogeneity (HOM), and Completeness (COM) scores. Figure 1a shows the spatial domain identification performance of different methods across Datasets 1–3. As shown, Banksy demonstrates the highest performance, followed closely by NichePCA. The identified spatial domains by different methods along with their ground truth annotation for a selected sample of Dataset 1 are shown in Fig. 1b. An ablation experiment further validated that NichePCA combines minimal complexity with maximal performance within its algorithmic framework (Supplementary Fig. S1).

**Figure 2.**
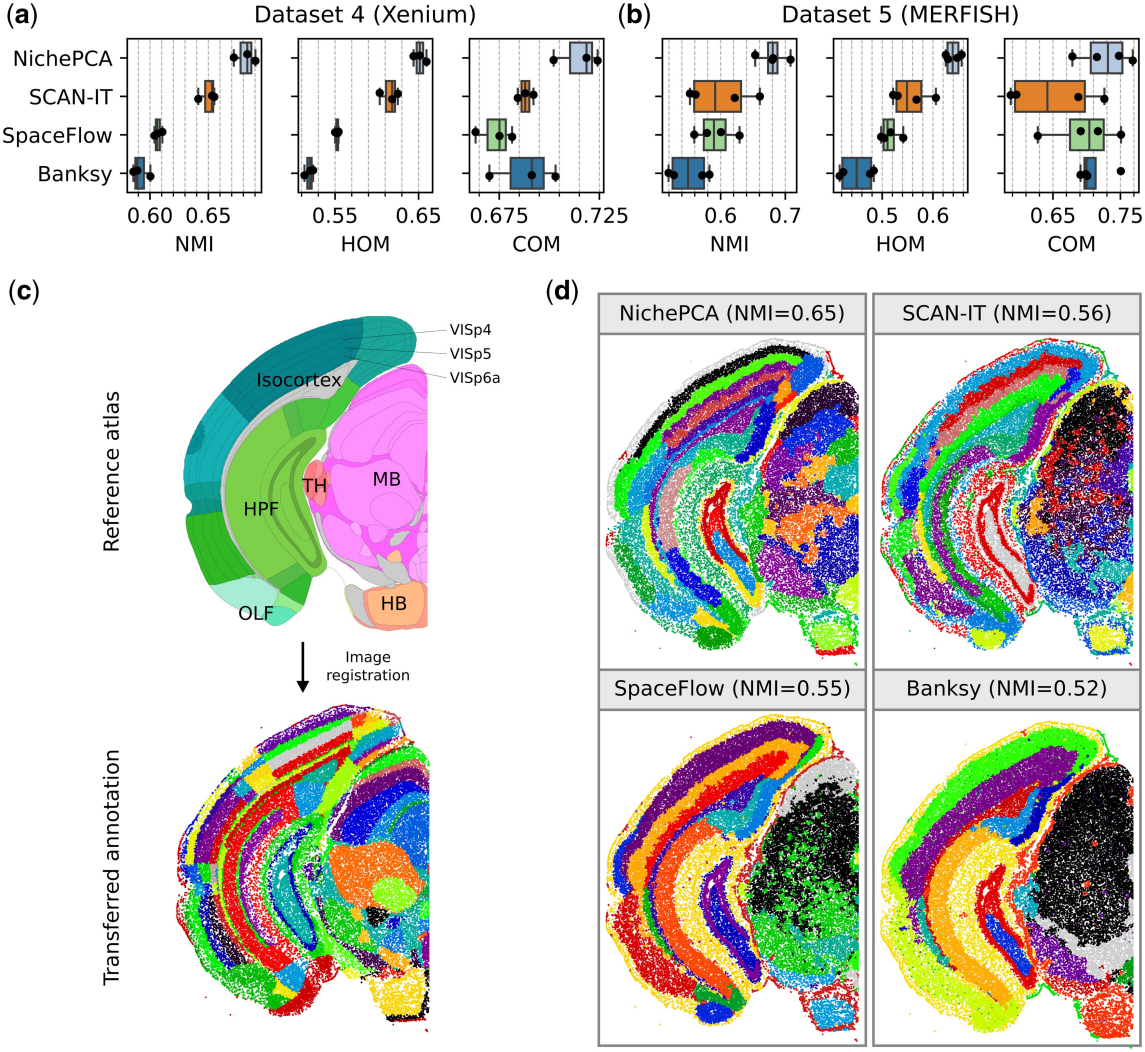
Quantitative performance evaluation on data beyond 80 000 cells per sample (Datasets 4 and 5). (a and b) Performance of NichePCA, SCAN-IT, SpaceFlow, and Banksy on Datasets 4 and 5, respectively, in terms of NMI, HOM, and COM scores. The dots show the performance per sample. We optimized the hyperparameters of each method across both datasets to ensure a fair comparison. (c) (top) Schematic domain annotation taken from the Allen Brain Reference Atlas (Wang et al. 2020) with labeled high-level areas. We additionally highlight some fine-grained domains inside the visual cortex. (bottom) Our ground truth domain annotations after image registration (see Section 2) projected onto the cell positions within one half of a selected sample of Dataset 5 (MERFISH). We only show color codings but omit explicit cluster labels to preserve readability. Cells that could not be assigned to any domain have been removed. All metrics for this sample are calculated using the resulting fine-grained cluster assignments. (d) Domain identification results for NichePCA, SCAN-IT, SpaceFlow, and Banksy on the same sample as in (c) with corresponding NMI scores in brackets. The NMI scores are calculated on both halves of the brain sample using the most fine-grained ground truth annotations.

While Datasets 1–3 contain <6000 cells per sample, state-of-the-art spatial sequencing technology can feature more than 100 000 cells. This recent technological improvement, coupled with the increased performance of recent image registration and cell segmentation workflows, can significantly improve the quality and quantity of information that can be retrieved from ST data (see Section 2). To understand the domain identification performance on state-of-the-art ST data, we next evaluated the four top-performing methods on two recent 10× Xenium (Dataset 4) and MERFISH (Dataset 5) mouse brain datasets, using recent registration and segmentation technology (see Section 2). In total, these datasets contain seven samples averaging about 100 000 cells per sample (Supplementary Tables S1 and S2). Ground truth domain annotations for Dataset 4 were taken from Bhuva *et al.* (2024). For Dataset 5, we generated ground truth domain annotations by registering annotations from the Allen Brain Reference Atlas (Wang *et al.* 2020) (see Section 2). Since we were not able to execute BASS (Li and Zhou 2022) and SpatialPCA (Shang and Zhou 2022) within a memory budget of 128 GB on any of the samples, we instead included SpaceFlow (Ren *et al.* 2022), the next-best algorithm, in this comparison. As shown in Fig. 2a and b, NichePCA significantly outperforms current state-of-the-art methods in all metrics on Datasets 4 and 5. Interestingly, Banksy, the best-performing method on Datasets 1 to 3, performs rather poorly on the more recent Datasets 4 and 5. On Dataset 4 NichePCA achieved a median NMI of 0.68, HOM of 0.65, and COM of 0.72, surpassing Banksy’s median NMI by 16, HOM by 25, and COM by 4 percentage points. For Dataset 5, NichePCA achieved a median NMI of 0.68, HOM of 0.64, and COM of 0.73, surpassing Banksy’s median NMI by 24, HOM by 42, and COM by 5 percentage points. We found this performance gap to persist when considering only transcripts inside the nuclei for Dataset 4, indicating that NichePCA’s superior performance is robust to changes in the cell segmentation procedure (Supplementary Fig. S2). The ground truth annotations and domain identification results of a selected sample from Dataset 5 are shown in Fig. 2c and d, respectively. NichePCA successfully identifies the separate layers in the cortex and even distinguishes different functional areas within these layers. A similar pattern can be observed for Dataset 4 (Supplementary Fig. S3).

**Figure 3.**
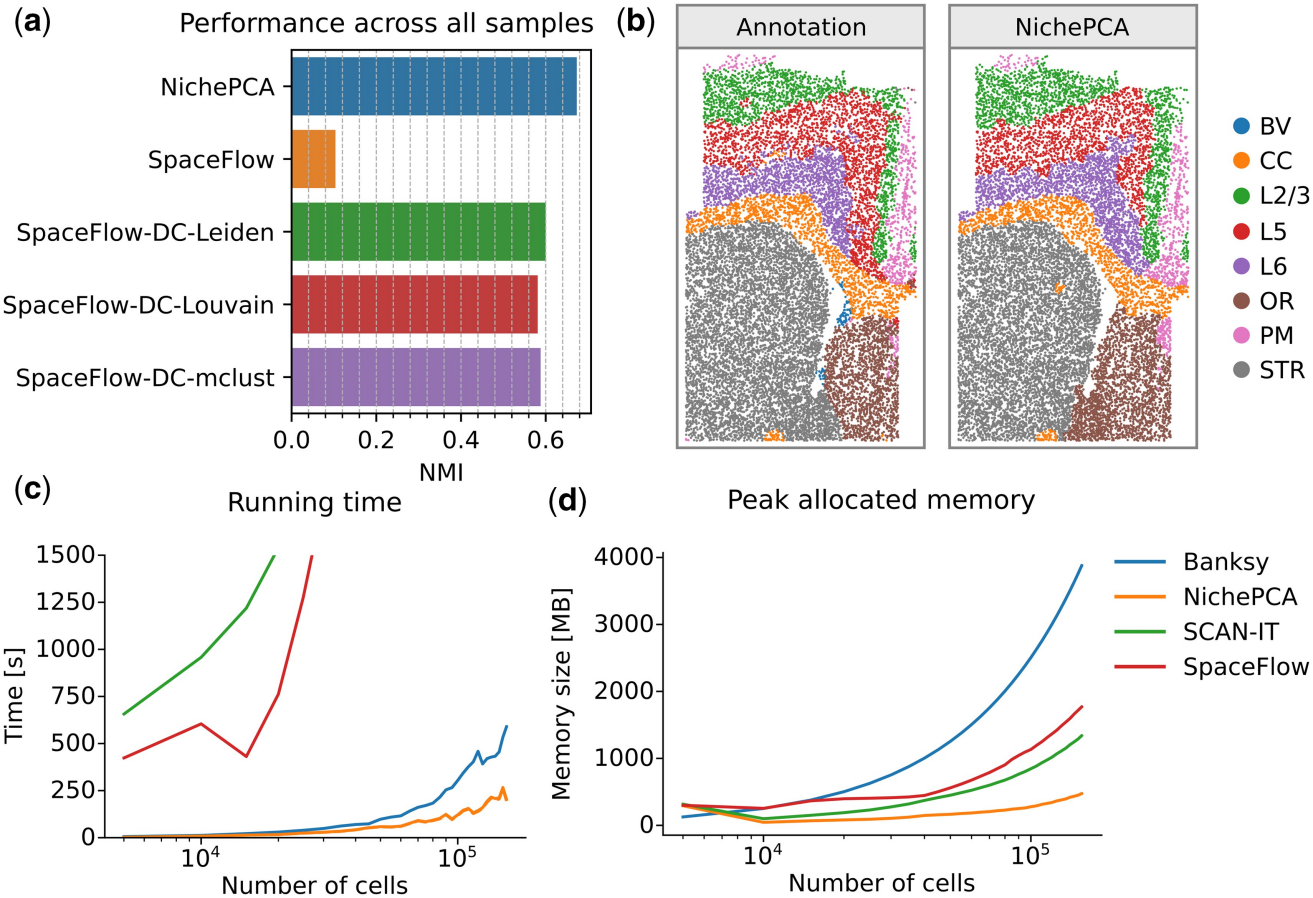
Multi-sample domain identification performance and scalability analysis. (a) Spatial domain identification performance across all 31 samples of Dataset 6 in terms of NMI score for NichePCA, SpaceFlow, and different variants of SpaceFlow, that have been developed in Yuan *et al.* (2024). All results apart from the ones for NichePCA are adopted from Yuan *et al.* (2024). Since the NMI score is calculated across all samples, cluster labels for the same spatial domain must be preserved across samples to achieve high scores. (b) Exemplary domain identification results for NichePCA on a selected sample of Dataset 6 and its corresponding ground truth annotations. Abbreviations for the different annotated domains are provided in the color legend. We manually matched the color coding of the NichePCA clusters to that of the ground truth domains. (c, d) Peak allocated memory in megabytes (MB) and running time in seconds on subsampled versions (5000–155 000 cells) of a sample from Dataset 4 for NichePCA, Banksy, SCAN-IT, and SpaceFlow. For a fair comparison, all methods are run solely on CPU (see Section 2).

Many spatial domain identification algorithms do not support the integration of multiple samples. Recently, Yuan *et al.* combined SpaceFlow and Harmony (Korsunsky *et al.* 2019) to obtain coherent spatial domain clusters across multiple samples. NichePCA is naturally compatible with Harmony, as both rely on PCA, allowing for seamless multi-sample spatial domain identification. To assess this compatibility quantitatively we used the same MERFISH dataset as Yuan *et al.* [Allen *et al.* (2023), Dataset 6], containing 31 samples (see Section 2). NichePCA outperforms the workflow developed by Yuan *et al.* both on a per-sample level as well as on all samples combined (Supplementary Fig. S4a, Fig. 3a). For a selected sample, the identified spatial domains and corresponding ground truth annotations are shown in Fig. 3b.

To showcase the scalability of NichePCA beyond one million cells, we selected a dataset (Dataset 7) from the BICCN 2.0 atlas (Zhang *et al.* 2023), containing four sub-datasets (Datasets 7.1–7.4, see Section 2) with up to 1.5 million cells each (Supplementary Fig. S5a), and applied NichePCA using the same multi-sample domain identification workflow as for Dataset 6. Importantly, we also chose the same number of nearest neighbors as previously determined for Datasets 4 and 5 (see Section 2). NichePCA performs well for all datasets apart from Dataset 7.1 both on a per-sample level and across all samples (Supplementary Fig. S5b and c).

We further assessed the different workflows in terms of their running time and memory consumption (Fig. 3c and d). For this, we selected a sample of Dataset 4 and randomly subsampled it to different numbers of cells ranging from 5000 to 155 000. Then, we applied Banksy, SCAN-IT, SpaceFlow, and NichePCA to each subsampled data. We found that NichePCA is by far the fastest and most memory-efficient method. The measured clustering times show that NichePCA primarily consumes time during the Leiden clustering step (Supplementary Fig. S4b). This could be further optimized by utilizing GPU implementations of the Leiden algorithm (e.g. see the RAPIDS Graph documentation, https://docs.rapids.ai).

NichePCA inherits the advantages of PCA, such as the interpretation of each PC in relation to genes. We can assess the variance explained as well as the top genes contributing to the variation in the data. For instance, in the analysis of the MERFISH brain data (Dataset 5), the first PC explains approximately 40% of the total variance present in the data, and the top genes contributing to it include oligodendrocyte markers such as *Olig1* and *Sox8* (Supplementary Fig. S6a and b). Directly relating gene contribution to cluster definition may involve post-hoc analysis, such as differential gene expression between clusters. However, this does not explain how clusters relate to data structure and how they are defined in gene space. We can project clusters and their centroids to PCA space (Supplementary Fig. S6c). This allows us to identify the top genes contributing to each cluster by multiplying the PC coordinates of cluster centroids by the PCA feature loadings (see Section 2). As an illustration, we examine cluster 29, which corresponds to the hippocampus region CA1 stratum radiatum (CA1sr) (Supplementary Fig. S6d and e). CA1 neurons are critical for hippocampal-dependent memory formation. Sstr4, a gene involved in the selection of memory strategies (Gastambide *et al.* 2009), is the gene with the highest positive contribution to the cluster 29 centroid. *Gpr161*, a gene localized in the hippocampus CA1 region (Mukhopadhyay *et al.* 2013), is also among the top four genes (Supplementary Fig. S6f). Among the genes with the highest negative contribution are those with unspecific expression in the CA1 region, such as *Olig1*, a marker for mature oligodendrocytes (Supplementary Fig. S6g).

## 4 Conclusion

In summary, this work introduces NichePCA, a simple neighborhood embedding-based algorithm for spatial domain identification. It matches or surpasses the performance of current state-of-the-art methods and offers significant scalability advantages. Moreover, NichePCA’s simplicity allows for the easy interpretation of clustering results.

Our work aligns conceptually with several recent studies demonstrating that relatively simple methods can yield good performance in spatial domain identification. For instance, CellCharter (Varrone *et al.* 2024) utilizes SCVI (Lopez *et al.* 2018) to compute low-dimensional embeddings before the neighborhood aggregation step. BINARY (Lin *et al.* 2024) investigates the impact of simplifying model inputs through gene expression binarization and MENDER (Yuan 2024) focuses on generating low-dimensional representations of the cell type composition instead of the gene expression per neighborhood. SPIN (Maher *et al.* 2023) leverages smoothed gene expression for clustering and mitigates autocorrelation between adjacent cells by random subsampling of neighbors.

In this work, we took a distinct reductionist approach by summarizing and comparing the key steps within the current spatial domain identification paradigm (Supplementary Fig. S1). We identified NichePCA as the best-performing lean algorithm that leverages the strong parallels between single-cell and spatial clustering.

We believe NichePCA can serve as a quick and efficient first-stop solution for identifying spatial domains, similar to how cell-level PCA is used for cell-type identification. While we acknowledge that the space of possible methods following the neighborhood embedding paradigm is far from explored, our empirical results suggest that higher complexity within this paradigm does not necessarily lead to better performance.

## Supplementary Material

btaf005_Supplementary_Data

## Data Availability

All data is publicly available and was processed as described in Section 2.
