# Supplementary material for: PCA-based spatial domain identification with state-of-the-art performance
Source: Bioinformatics. 2025 Jan 7;41(1):btaf005. doi: 10.1093/bioinformatics/btaf005 (PMC11761416; doi:10.1093/bioinformatics/btaf005)
Supplement: btaf005_Supplementary_Data [file btaf005_supplementary_data.zip › btaf005_Supplementary_Data.zip!/8d8a8_NichePCA_supplementary_figures.pdf]

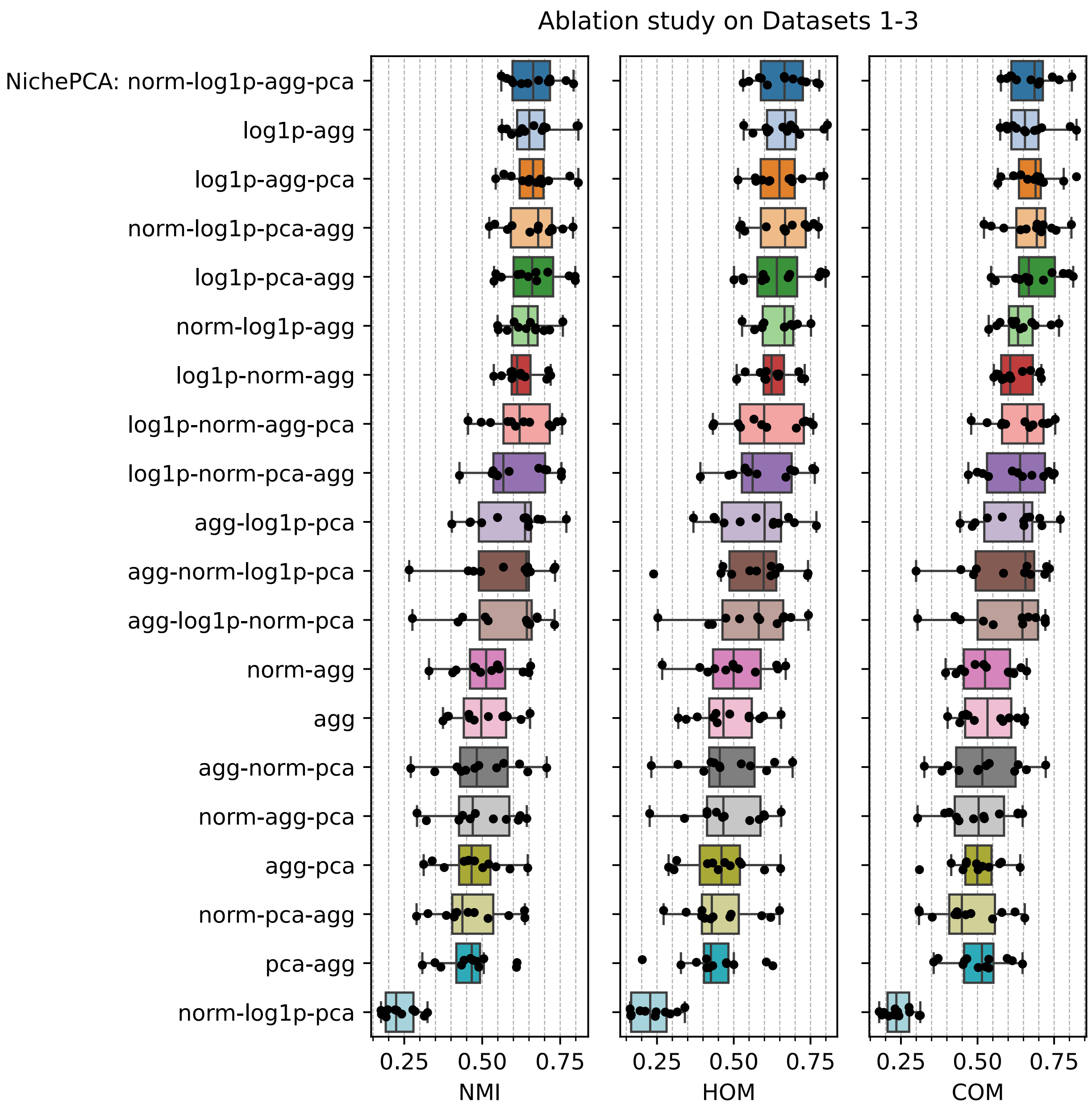

Supplementary Figure 1: Ablation results for different variants of NichePCA on Datasets 1-3 regarding NMI, HOM, and COM scores. The annotations represent the steps of the different variants as follows: "agg" - mean neighborhood aggregation, "norm" - normalization to sum to the median value per feature, "log1p" - log-transformation with pseudocount of 1, "pca" - principal component analysis. Dots indicate the performance per sample.

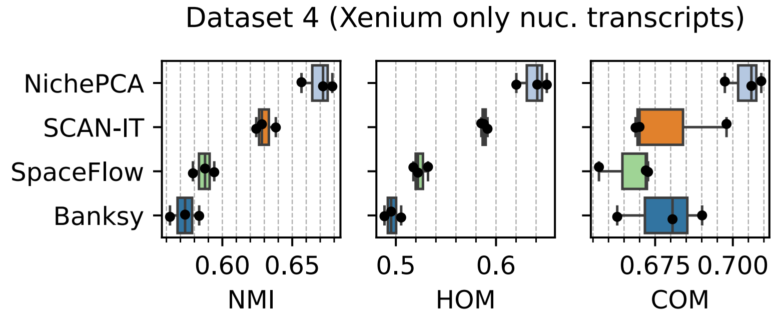

Supplementary Figure 2: Performance in terms of NMI, HOM, and COM scores (left to right) for NichePCA, Banksy, SCAN-IT, and SpaceFlow on Dataset 4 (Xenium), considering only transcripts located within the segmented nuclei. The dots indicate the performance per sample.

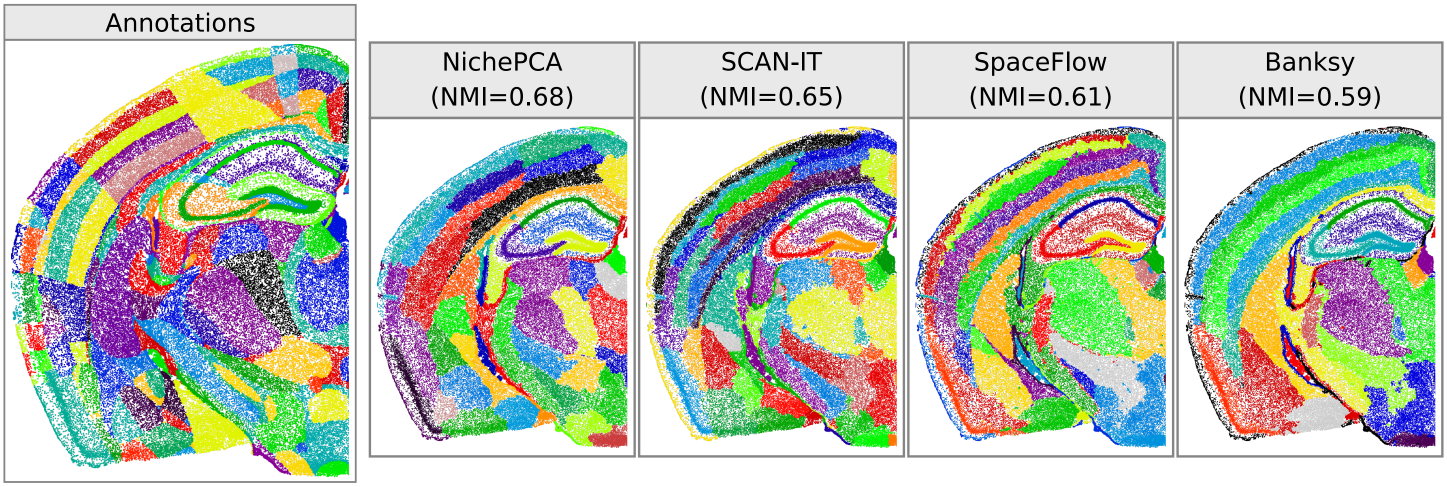

Supplementary Figure 3: Spatial domain identification results for NichePCA, SCAN-IT, SpaceFlow, and Banksy and ground truth annotations for one half of a selected sample of Dataset 4. The NMI scores for each method on this sample are shown in brackets and have been calculated on both halves of the brain sample.

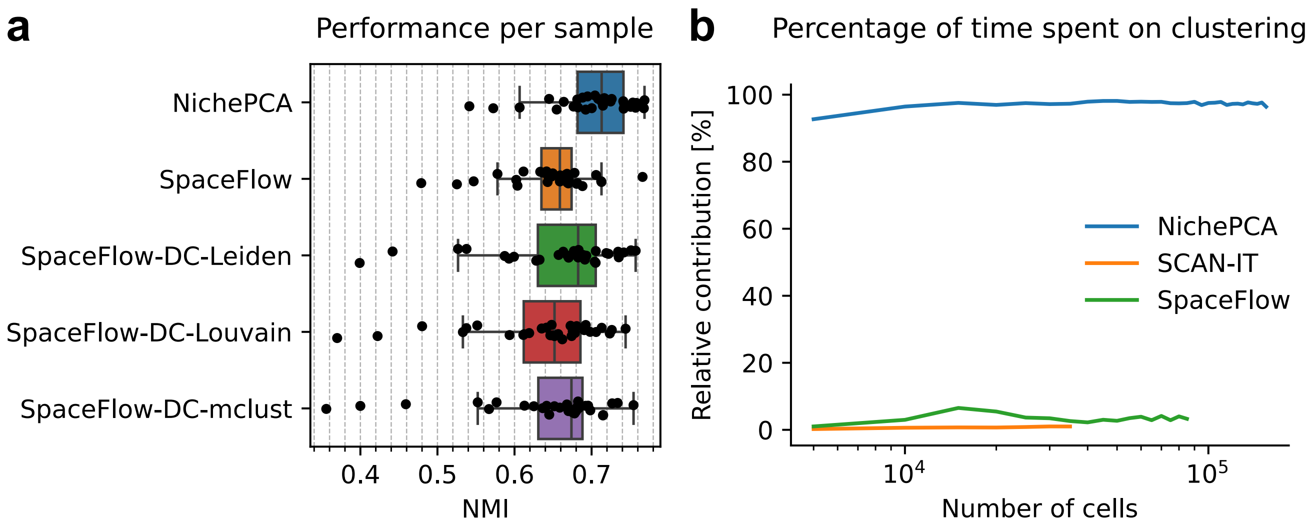

Supplementary Figure 4: a, Spatial domain identification performance for all individual samples of Dataset 6 in terms of NMI score. Each dot represents the performance on one sample. For SpaceFlow and its modifications we adopted the results from Yuan et al. (2024). b, Percentage of the total running time spent on clustering for NichePCA, SCAN-IT, and SpaceFlow. This evaluation is performed on the same sample of Dataset 4 and its subsampled versions as in Fig. 2c-d.

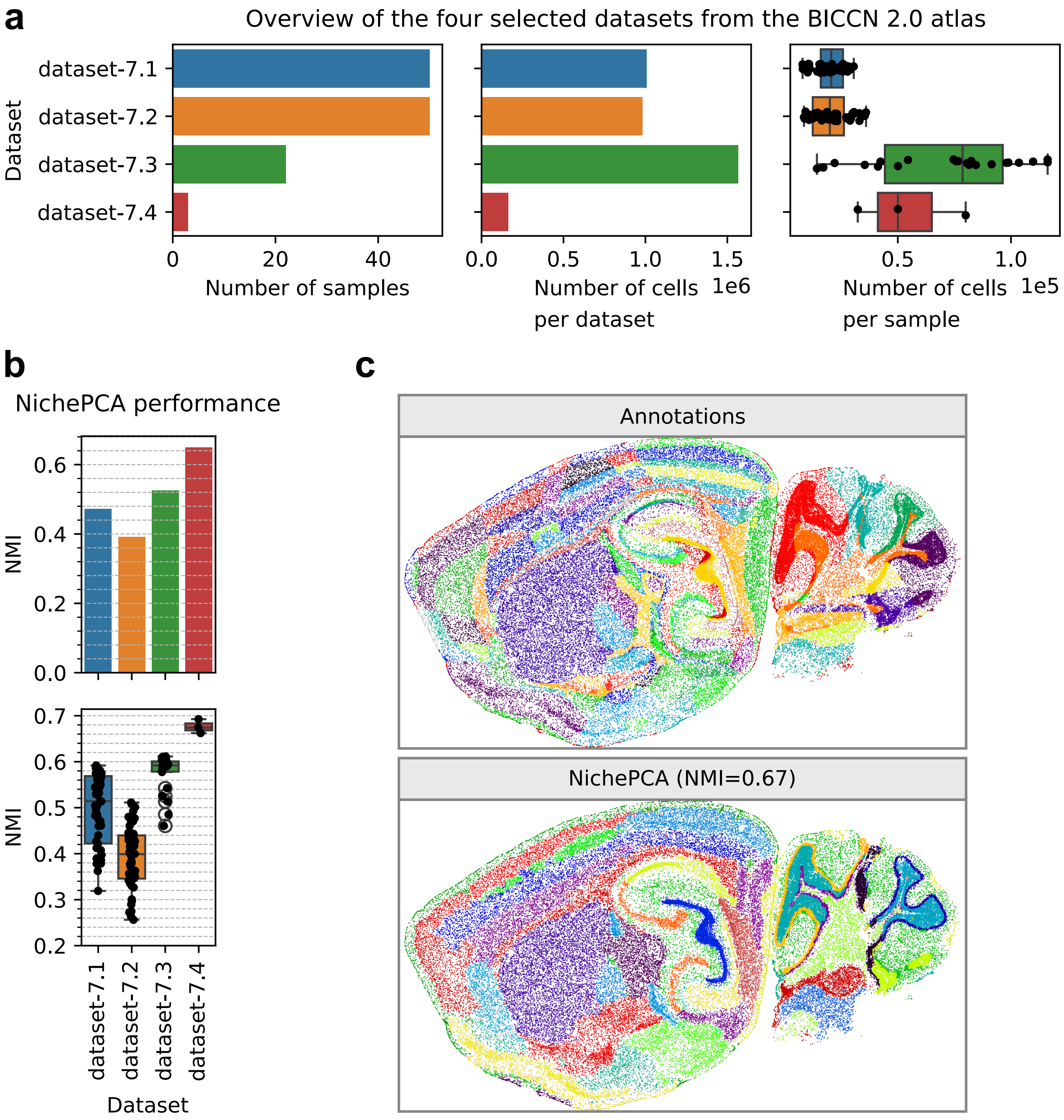

Supplementary Figure 5: Application of NichePCA to large-scale spatial transcriptomics data. a, Overview of the four selected datasets from the BICCN 2.0 atlas in terms of the number of samples (left), the number of cells per dataset (middle), and the number of cells per sample (right). For the Datasets 7.1 and 7.2, we restricted our analysis to a random subset of 50 samples. b, Performance of NichePCA on the four selected datasets in terms of NMI. We used NichePCA to identify coherent spatial domains across all samples of each dataset as for Dataset 6. We analyzed the performance both on a dataset level (top) as well as on a per-sample level (bottom). c, Exemplary domain identification results for NichePCA on a selected sample of Dataset 7.4 and its corresponding ground truth annotations.

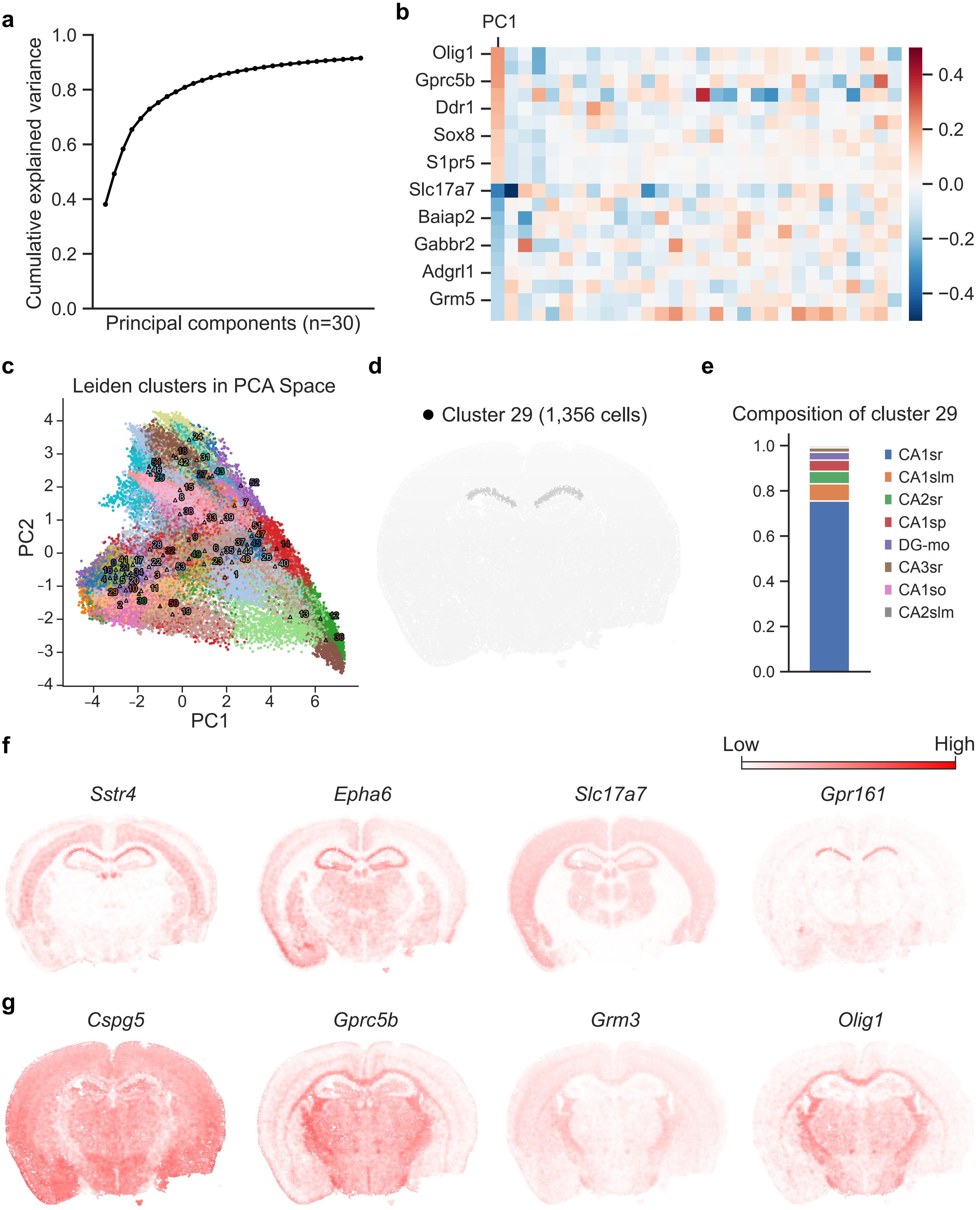
a
Cumulative explained variance
Principal components (n=30)
b
PC1
Olig1
Gprc5b
Ddr1
Sox8
S1pr5
Slc17a7
Baiap2
Gabbr2
Adgrl1
Grm5
c
Leiden clusters in PCA Space
PC2
PC1
d
Cluster 29 (1,356 cells)
e
Composition of cluster 29
CA1sr
CA1slm
CA2sr
CA1sp
DG-mo
CA3sr
CA1so
CA2slm
f
Low
High
Sstr4
Epha6
Slc17a7
Gpr161
g
Cspg5
Gprc5b
Grm3
Olig1

Supplementary Figure 6: a, Cumulative variance explained by each principal component for a selected sample of Dataset 5. b, Loadings of genes with the most contribution to the first principal components. The five most positive and the five most negative genes are shown. c, Scatter plot showing the first and second principal components. The cells are colored by their cluster and the centers are labeled. d, Spatial localization of cells in cluster 29. e, Barplot showing the proportion of the ground truth domains within cluster 29. f, g, Expression patterns of genes with the most positive (f) and negative (g) contributions to the centroid of cluster 29.
